# Development of mismatch amplification mutation assays for the differentiation of MS1 vaccine strain from wild-type *Mycoplasma synoviae* and MS-H vaccine strains

**DOI:** 10.1371/journal.pone.0175969

**Published:** 2017-04-18

**Authors:** Zsuzsa Kreizinger, Kinga Mária Sulyok, Dénes Grózner, Katinka Bekő, Ádám Dán, Zoltán Szabó, Miklós Gyuranecz

**Affiliations:** 1 Institute for Veterinary Medical Research, Centre for Agricultural Research, Hungarian Academy of Sciences, Budapest, Hungary; 2 Veterinary Diagnostic Directorate, National Food Chain Safety Office, Budapest, Hungary; 3 Intervet Hungária Kft., part of MSD Animal Health, Budapest, Hungary; Universidad Nacional de la Plata, ARGENTINA

## Abstract

*Mycoplasma synoviae* is an economically significant pathogen in the poultry industry, inducing respiratory disease and infectious synovitis in chickens and turkeys, and eggshell apex abnormality in chickens. Eradication, medication and vaccination are the options for controlling *M*. *synoviae* infection. Currently there are two commercial, live, attenuated vaccines available against *M*. *synoviae*: the temperature sensitive MS-H vaccine strain and the NAD independent MS1 vaccine strain. Differentiation of vaccine strains from field isolates is essential during vaccination and eradication programs. The present study provides melt-curve and agarose gel based mismatch amplification mutation assays (MAMA) to discriminate the MS1 vaccine strain from the MS-H vaccine strain and wild-type *M*. *synoviae* isolates. The assays are based on the A/C single nucleotide polymorphism at nt11 of a HIT family protein coding gene. The melt- and agarose-MAMAs reliably distinguish the MS1 vaccine strain genotype from the MS-H vaccine strain and wild-type *M*. *synoviae* isolate genotype from 10^2^ template number/DNA sample. No cross-reactions with other avian *Mycoplasma* species were observed. The assays can be performed directly on clinical samples and they can be run simultaneously with the previously described MAMAs designed for the discrimination of the MS-H vaccine strain. The developed assays are applicable in laboratories with limited facilities and promote the rapid, simple and cost effective differentiation of the MS1 vaccine strain.

## Introduction

Infection by *Mycoplasma synoviae* occurs worldwide leading to respiratory diseases and synovitis in chickens and turkeys, and eggshell apex abnormality in chickens, and as a consequence to high economic losses in the poultry industry [1–3]. The severity of clinical signs can vary from sub-clinical to severe forms and it depends on the pathogenicity of the agent and on the presence of other bacteria or viruses (e.g. *Escherichia coli* or infectious bronchitis virus) [1–2,4]. *M*. *synoviae* can be transmitted horizontally, colonizing first the respiratory tract or vertically through the eggs [4–6]. To prevent the introduction of *M*. *synoviae* into a flock effective biosecurity programs should be applied and mycoplasma-free sources should be used for restocking. Medication can reduce the economic losses in the infected flocks and inhibit the transmission through the eggs [6]. Vaccination is another option to control the disease, especially in commercial layers [6–7]. At present, two commercial attenuated live vaccines are available against *M*. *synoviae*: the temperature sensitive (ts^+^) MS-H vaccine strain (Vaxsafe® MS, Bioproperties Pty Ltd.) which was developed by the chemical mutagenesis of an Australian field strain (86079/7NS); and the NAD independent MS1 vaccine strain (Nobilis® MS Live, MSD Animal Health Inc.) which was obtained spontaneously during *in vitro* passages of the type strain (WVU 1853, ATCC 25204, NCTC 10124) [8–9]. Differentiation of vaccine and wild strains is crucial in the control programs. Several molecular techniques are available for the discrimination of the ts^+^ MS-H strain, its ts^-^ re-isolates and field isolates, such as the sequence analysis of the *vlhA* gene or identification of specific point mutations in the *obg* gene by high resolution melting-curve (HRM) analysis or mismatch amplification mutation assays (MAMAs) [10–12]. The comparison of the whole genomes of the WVU 1853 parent strain and the MS1 vaccine strain revealed a single nucleotide polymorphism (SNP) in the histidine triad motif (HIT) family protein coding gene [9]. The present study provides melt-curve and agarose-gel based MAMAs specific for the SNP in the HIT family protein coding gene to discriminate MS1 vaccine and field strains. The assays were developed to be used simultaneously with the previously described MS-H1 and MS-H2 MAMAs [12], hence differentiating the MS1 vaccine, ts^+^ MS-H vaccine, ts^-^ MS-H vaccine re-isolate and field strains in one simple and cost-efficient step.

## Methods

### Samples

The vaccine strains MS1 (Nobilis® MS Live) and the ts^+^ MS-H (Vaxsafe® MS-H) used during the examinations originated from their commercial distributors. *M*. *synoviae* (WVU 1835, ATCC 25204, NCTC 10124) type strain was used as control in the assays. *M*. *anatis* (ATCC 25524), *M*. *anseris* (ATCC 49234), *M*. *cloacale* (ATCC 35276), *M*. *columbinasale* (ATCC 33549), *M*. *columbinum* (ATCC 29257), *M*. *columborale* (ATCC 29258), *M*. *gallinaceum* (ATCC 33550), *M*. *gallinarum* (ATCC 19708), *M*. *gallisepticum* (ATCC 19610), *M*. *gallopavonis* (ATCC 33551), *M*. *iners* (ATCC 19705), *M*. *iowae* (ATCC 33552) and *M*. *meleagridis* (NCTC 10153) type strains and a *M*. sp. 1220 strain isolated by the authors were used for the examinations of cross-reactions. The *M*. sp. 1220 strain originated from a cloaca swab of a goose sampled during routine diagnostic examinations in 2016 in Csongrád county, Hungary.

A total of 27 bacterial strains (including a ts^-^ MS-H vaccine re-isolate and 26 *M*. *synoviae* wild-type strains) were collected and isolated by the authors during routine diagnostic examinations of live animals (Table 1). For the validation of the assays, clinical samples including trachea swabs, trachea tissue samples and sample collection cards (Whatman FTA card, Whatman, Maidstone, UK) were collected from vaccinated (160 chickens) and clinically infected (seropositive) chickens and turkeys (total of 59 animals; Table 1). Ethical approval and specific permission were not required for the study as all samples and strains were collected by the authors during routine diagnostic examinations with the consent of the owners. Trachea swabs originated from live animals, tissue samples and FTA cards originated from animals found dead in the farms. The study did not involve endangered or protected animals.

**Table 1:** Background information and genotype of the *Mycoplasma synoviae* strains and clinical samples included in this study.

Sample ID^a^	Sample type	Host^b^	Age of host (weeks)	Type of host	Origin of sample^c^	Year	Gt^d^	Reference
MS1v	MS1 vaccine strain				Nobilis® MS Live, MSD Animal Health		MS1	
MS ref	*M*. *synoviae* type strain				WVU 1853, ATCC25204, NCTC10124		Wt	
ts^+^ MS-H	ts^+^ MS-H vaccine strain				Vaxsafe® MS, Bioproperties Pty Ltd.		Wt	
ts^-^ MS-H	ts^-^ MS-H vaccine re-isolate	ch	73	layer	farm1, Jász-Nagykun-Szolnok, Hungary	2015	Wt	
MS 1	*M*. *synoviae* strain	ch			USA	1990	Wt	K3009/70 [10]
MS 2	*M*. *synoviae* strain	ch			Slovenia	2002	Wt	ULB02/T6 [13]
MS 3	*M*. *synoviae* strain	ch			Slovenia	2008	Wt	ULB08/T3 [14]
MS 4	*M*. *synoviae* strain	ch	36	layer	farm2, Nógrád, Hungary	2014	Wt	
MS 5	*M*. *synoviae* strain	ch	35	breeder	farm3, Veszprém, Hungary	2015	Wt	
MS 6	*M*. *synoviae* strain	ch	38	breeder	farm4, Jász-Nagykun-Szolnok, Hungary	2015	Wt	
MS 7	*M*. *synoviae* strain	ch	7	broiler	farm5, Komárom-Esztergom, Hungary	2015	Wt	
MS 8	*M*. *synoviae* strain	ch	65	layer	farm6, Borsod-Abaúj-Zemplén, Hungary	2015	Wt	
MS 9	*M*. *synoviae* strain	ch	47	breeder	farm7, Zala, Hungary	2015	Wt	
MS10	*M*. *synoviae* strain	ch	64	layer	farm8, Pardubice, Czech Republic	2015	Wt	
MS 11	*M*. *synoviae* strain	ch	28	layer	farm9, Oryol, Russia	2015	Wt	
MS 12	*M*. *synoviae* strain	ch	4	layer	farm10, Cherkasy, Ukraine	2015	Wt	
MS 13	*M*. *synoviae* strain	ch	48	layer	farm11, Fejér, Hungary	2016	Wt	
MS 14	*M*. *synoviae* strain	ch	30	layer	farm12, South Moravia, Czech Republic	2016	Wt	
MS 15	*M*. *synoviae* strain	ch	45	breeder	farm13, Serbia	2016	Wt	
MS 16	*M*. *synoviae* strain	t			USA	1983	Wt	K1968/clone ZC3 [10]
MS 17	*M*. *synoviae* strain	t	16	broiler	farm14, Győr-Moson-Sopron, Hungary	2014	Wt	
MS 18	*M*. *synoviae* strain	t	14	broiler	farm15, Vas, Hungary	2014	Wt	
MS 19	*M*. *synoviae* strain	t	11	broiler	farm16, Győr-Moson-Sopron, Hungary	2014	Wt	
MS 20	*M*. *synoviae* strain	t	13	broiler	farm17, Békés, Hungary	2015	Wt	
MS 21	*M*. *synoviae* strain	t	13	broiler	farm18, Komárom-Esztergom, Hungary	2016	Wt	
MS 22	*M*. *synoviae* strain	t	16	broiler	farm19, Győr-Moson-Sopron, Hungary	2016	Wt	
MS 23	*M*. *synoviae* strain	t	17	broiler	farm20, Tolna, Hungary	2016	Wt	
MS 24	*M*. *synoviae* strain	t	20	broiler	farm21, Veszprém, Hungary	2016	Wt	
MS 25	*M*. *synoviae* strain	t	19	broiler	farm22, Veszprém, Hungary	2016	Wt	
MS 26	*M*. *synoviae* strain	t	13	broiler	farm23, Somogy, Hungary	2016	Wt	
TI 1	tissue from infected animal	ch	48	breeder	farm24, Pest, Hungary	2016	Wt	
TI 2	tissue from infected animal	ch	34	layer	farm25, Ternopil, Ukraine	2016	Wt	
FI 1	FTA card from infected animal	ch	34	layer	farm25, Ternopil, Ukraine	2016	Wt	
FI 2	FTA card from infected animal	ch	40	layer	farm25, Ternopil, Ukraine	2016	Wt	
SI 1–5 (1)	swabs from infected animals	ch	54	breeder	farm26, Pest, Hungary	2015	Wt	
SI 6–10 (1)	swabs from infected animals	ch	25	layer	farm27, Borsod-Abaúj-Zemplén, Hungary	2016	Wt	
SI 11–15 (1)	swabs from infected animals	ch	30	layer	farm28, Hajdú-Bihar, Hungary	2016	Wt	
SI 16–20 (1)	swabs from infected animals	ch	24	layer	farm29, Oryol, Russia	2016	Wt	
SI 21–25 (1)	swabs from infected animals	t	47	layer	farm30, Vas, Hungary	2016	Wt	
SI 26–30 (1)	swabs from infected animals	t	20	broiler	farm35, Győr-Moson-Sopron, Hungary	2016	Wt	
SI 30–35 (1)	swabs from infected animals	t	18	broiler	farm16, Győr-Moson-Sopron, Hungary	2016	Wt	
SI 36–40 (1)	swabs from infected animals	t	11	broiler	farm36, Hajdú-Bihar, Hungary	2016	Wt	
SI 41–45 (1)	swabs from infected animals	t	18	broiler	farm37, Komárom-Esztergom, Hungary	2016	Wt	
SI 46–50 (1)	swabs from infected animals	t	16	broiler	farm38, Burgenland, Austria	2016	Wt	
SI 51–55 (1)	swabs from infected animals	t	17	broiler	farm39, Burgenland, Austria	2016	Wt	
SV 1–30 (6)	swabs from vaccinated animals	ch	28	breeder	farm3, Veszprém,Hungary	2016	MS1	
SV 31–70 (8)	swabs from vaccinated animals	ch	29	breeder	farm40, Veszprém,Hungary	2016	MS1	
SV 71–100 (6)	swabs from vaccinated animals	ch	14	breeder	farm41, Veszprém,Hungary	2016	MS1	
SV 100–160 (12)	swabs from vaccinated animals	ch	28	breeder	farm42, Baranya, Hungary	2016	MS1	

^a^DNA from swab samples were gained in DNA pools (each pool contained 5 swabs); numbers in brackets represent the number of DNA pools examined

^b^all samples were collected from the trachea of the animals; ch: chicken, t: turkey

^c^farm, region and country of origin of the samples

^d^Gt: genotype according to the MS1 DIVA-test; Wt: wild-type, MS1: MS1 vaccine, -: negative

DNA was extracted from the strains, trachea swabs (in DNA pools, each containing 5 trachea swabs), tissue samples and FTA cards with the Qiamp DNA Mini kit (Qiagen GmbH, Hilden, Germany). The ts^-^ MS-H re-isolate was identified by the MS-H1 and MS-H2 MAMAs, based on the point mutations at nt367 (G) and nt629 (C) in the *obg* gene [12].

### Sequence analysis

**Fig 1:**
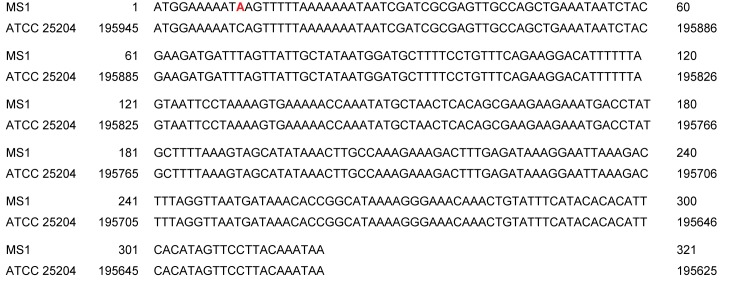
Sequence alignment of the HIT family protein coding gene of the MS1 vaccine strain and its parent strain ATCC 25204. Nucleotide polymorphism at 11. position (red) results in premature stop codon in strain MS1.

The HIT family protein coding gene of the MS1 strain was amplified by conventional PCR using the primers MS-HL1 (5’–ACT GTA AAT GAC GCC TTT TCT AC– 3’) and MS-HL2 (5’–ACC GCT TAT GCA AGT AAA TTA TT– 3’), designed in the present study. The reaction mixture contained 5 μl 5X Green GoTaq Flexi Buffer (Promega Inc., Madison, WI), 2.5 μl MgCl_2_ (25mM, Promega), 0.5 μl dNTP (10 mM, Qiagen Inc., Valencia, CA), 1 μl of each primer (10 pmol/μl), 0.25 μl GoTaq DNA polymerase (5 U/μl; Promega) and 2 μl of target DNA in a total volume of 25 μl. Thermocycling parameters consisted of 95°C for 2 min, then 40 cycles of 95°C for 30sec, 55°C for 30 sec and 72°C for 1 min and a final elongation step at 72°C for 5 min. The amplicons were visualised in 1% agarose gel (Seakem Agarose, Lonza Group Ltd., Basel, Switzerland) under UV light. The amplicons were extracted from the gel using the QIAquick Gel Extraction kit (Qiagen) and submitted for Sanger sequencing on an ABI 3700 DNA Analyzer (Applied Biosystems, Foster City, CA). Sequence of the amplicon of the MS1 strain (660 bp) contains the complete sequence of the HIT family protein coding gene (321 bp) and fragments of the flanking regions (GenBank accession number: KY712765). Alignment of the sequences of the HIT family protein coding genes of the *M*. *synoviae* type strain (ATCC 25204, GenBank Accession Number: CP011096, region (complement): 195625–195945) and the MS1 strain (KY712765) revealed a point mutation at nucleotide (nt) 11 in accordance with the previous publication [9]. The described TCA (Ser) / TAA (stop codon) polymorphism results a stop codon in the MS1 vaccine strain (Fig 1).

### Development of mismatch amplification mutation assays

MAMA is based on allele-specific competing primers and is used widely for SNP detection [15]. The allele-specific primers are SNP specific at the 3’end and a single base mismatch at the ante-penultimate (-3) position enhances the SNP discrimination capacity of the assay. A 15-20bp GC-clamp is added at the 5’end to one of the allele-specific primers which increases the melting temperature (T_m_) and the size of the resulting PCR product. The difference between the T_m_ and the product size is detectable by fluorescent dye on a real-time PCR platform (melt-MAMA) or by 3% agarose gel electrophoresis (agarose-MAMA), respectively.

In the current study melt- and agarose-MAMAs were developed to identify the point mutation at nt11 in the HIT family protein coding gene of *M*. *synoviae* (GenBank Accession Number: CP011096, region (complement): 195625–195945). Genome location, primer sequences, annealing and melting temperatures for the assays and PCR product sizes can be found in Table 2. The assays were optimised by altering primer ratios [15]. The MAMAs were designed to be used concurrently with the previously described MS-H1 and MS-H2 melt- and agarose-MAMAs [12].

**Table 2:** SNP locations in the HIT family protein coding gene, SNP state, primer sequences, primer volumes, annealing temperature (T_a_), melting temperatures (T_m_) and PCR product sizes for the MS1 melt- and agarose-MAMAs.

SNP position	SNP state	MAMA primer names	MAMA primer sequences	Primer (10 pmol/μl) volume in 10 μl reaction mixture (μl)		T_a_ (°C)	T_m_ in °C (95% CI)	Product size (bp)
				melt	agarose			
11	C/A	MS1	ggggcggggcggggcgATTTTGTTGCAAAATAATGGAAAAcTa	0.150	1	58	79.1 (78.9–79.3)	83
		MSW	ATTTTGTTGCAAAATAATGGAAAAtTC	0.150	8		73.5 (73.3–73.8)	67
		MSR	TTTCAGCTGGCAACTCGCG	0.150	1			

Melt-MAMA PCR reactions were performed in 10 μl total volume, containing 1μl target DNA diluted in 2 μl 5X Color-less GoTaq Flexi Buffer (Promega), 1 μl MgCl_2_ (25mM), 0.3 μl dNTP (10 mM, Qiagen), 0.5 μl EvaGreen (Biotium Inc., Hayward, CA), primers (10 pmol/μl) according to Table 1 and 0.08 μl GoTaq DNA polymerase (5 U/μl; Promega). Melt-MAMAs were carried out on an Applied Biosystems Step-One Plus real-time PCR system with StepOne Software^TM^ v2.2.2 and on Qiagen Rotor-Gene Q real-time PCR system, software version 2.3.1. Thermocycling parameters were 95°C for 10 min, followed by 39 cycles of 95°C for 15 sec and 58°C for 1 min and a dissociation protocol comprising 95°C for 15 sec, followed by incremental temperature ramping (0.2°C) from 58°C to 95°C. EvaGreen fluorescent intensity was measured at 525 nm at each ramp interval and plotted against temperature.

Agarose-MAMA PCR reaction mixtures contained 2 μl target DNA diluted in 5 μl 5X Green GoTaq Flexi Buffer (Promega), 2.5 μl MgCl_2_ (25mM), 0.5 μl dNTP (10 mM, Qiagen), primers (10 pmol/μl) according to Table 1 and 0.25 μl GoTaq DNA polymerase (5 U/μl; Promega). Agarose-MAMAs were performed in a C1000™ Touch Thermal Cycler (Bio-Rad Laboratories, Inc., Berkeley, CA, USA). Thermocycling parameters for the agarose-MAMA were 94°C for 5 min, followed by 40 cycles at 94°C for 30 sec, 58°C for 30 sec and 72°C for 30 sec with a final elongation step at 72°C for 5 min. Electrophoresis was performed in 3% agarose gel (MetaPhor Agarose, Lonza Group) and a 20-bp DNA ladder (O'Range Ruler 20 bp, Thermo Fisher Scientific Inc.) was used as molecular weight marker.

### Validation of mismatch amplification mutation assays

In order to test the intra-run repeatability at least four replicates of the two genotypes (wild-type and MS1 vaccine-type) were examined within the same run. For inter-run reproducibility test, the duplicate of at least ten samples from each genotype was examined in separate tests.

**Fig 2:**
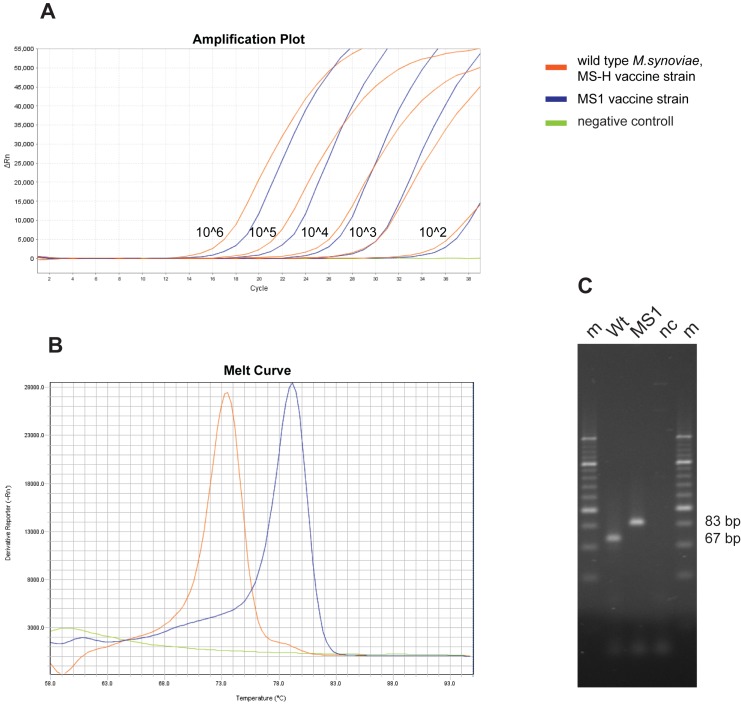
Amplification plot, melting-curves and PCR product sizes of MS1 melt- and agarose-MAMAs. Amplification plot (**A**) of dilution series of the MS1 vaccine strain (blue lines) and the *M*. *synoviae* type strain (WVU 1853, ATCC 25204, NCTC 10124; orange lines) showing the sensitivity of the assay. Green line represents negative control. Melting curves (**B**) show melting temperatures for the MS1 vaccine strain (T_m_: 79.3°C; blue line) and wild-type or MS-H vaccine strain (T_m_: 73.6°C; orange line). Electrophoresis (**C**) was performed in 3% agarose gel (MetaPhor Agarose, Lonza Group Ltd., Basel, Switzerland) and a 20-bp DNA ladder (O'Range Ruler 20 bp, Thermo Fisher Scientific Inc.) was used as molecular weight marker (m). The MS1 vaccine strain (MS1) yielded 83 bp fragment, while the wild-type or MS-H vaccine strain (Wt) produced 67 bp fragments.

In order to test the sensitivity of the assays, 10 fold dilutions in replicates of four of the MS1 vaccine and *M*. *synoviae* type strain (WVU 1853, ATCC 25204, NCTC 10124) were used (Fig 2). Template copy number of the strains was calculated from the amount of DNA using the online tool developed by Staroscik [16]. The specificity of the assays was tested by including the following avian *Mycoplasma* species in the analysis: *M*. *anatis*, *M*. *anseris*, *M*. *cloacale*, *M*. *columbinasale*, *M*. *columborale*, *M*. *gallinaceum*, *M*. *gallinarum*, *M*. *gallisepticum*, *M*. *gallopavonis*, *M*. *iners*, *M*. *iowae*, *M*. *meleagridis* and *M*. sp. 1220. Also, the assays were performed on clinical samples originating from *M*. *synoviae* vaccinated and infected animals. The assays were considered valid if both allelic DNA templates were genotyped accurately and consistently under the same conditions [15].

## Results

The developed melt- and agarose-MAMAs clearly identified the A-C polymorphism at the nt11 of the HIT family protein coding gene, distinguishing the wild, virulent *M*. *synoviae* strains and the MS-H vaccine strain from the MS1 vaccine strain, respectively. Due to the GC-clamp at the 5’ end of the primer specific for the MS1 vaccine strain genotype, the melting temperature in the melt-MAMA for the MS1 vaccine genotype (T_m_: 79.1°C, CI95: 78.9–79.3°C on Step-One system and 79.3°C, CI95: 79.2–79.5°C on Rotor-Gene system) is higher by approximately 5°C than for the wild-type or MS-H vaccine genotype (T_m_: 73.5°C, CI95: 73.3–73.8°C on Step-One system and 74°C, CI95: 73.9–74.2°C on Rotor-Gene system). For the same reason, the PCR product size in the agarose-MAMA for the MS1 vaccine genotype (83 bp) is longer by 16 bp than for the wild-type or MS-H vaccine genotype (67 bp) (Table 2, Fig 2).

Consistent allele calls were observed by melt-MAMA until as low as 10^2^ template number displaying a Ct range of 31–35. Negative controls or templates of other avian *Mycoplasma* species either were not amplified or generated non-specific products (with Ct above 37) with melt-profiles differing from the profiles of the expected two allelic states. Similarly, agarose-MAMAs worked reliable on samples containing 10^2^ template number and neither of the tested other avian *Mycoplasma* species caused cross-reaction (no amplicons observed). The *M*. *synoviae* type strain (WVU 1835, ATCC 25204, NCTC 10124), the ts^+^ MS-H vaccine strain (Vaxsafe® MS-H), the ts^-^ MS-H re-isolate, the 26 clinical *M*. *synoviae* isolates, the two tissue samples, the two FTA cards and the 11 DNA pools from the trachea swabs taken from clinically infected chickens and turkeys showed the wild-type strain profile while the MS1 vaccine strain (Nobilis® MS Live) and the 32 DNA pools from the trachea swabs taken from vaccinated chickens showed the MS1 vaccine strain profile in the melt- and agarose-MAMAs (Table 1). The results were confirmed by sequencing of the HIT family protein coding gene of these samples (data not shown).

## Discussion

*M*. *synoviae* has great impact on the poultry industry and vaccination is a feasible and cost effective option to reduce economic losses. However, the differentiation of infected from vaccinated animals (DIVA) is crucial in the vaccination programs, animal trading and eradication programs.

The live, attenuated vaccine strains currently available against *M*. *synoviae* are the ts^+^ MS-H vaccine (Vaxsafe® MS-H) and the MS1 vaccine (Nobilis® MS Live) strains. The MS-H vaccine strain was developed by chemical mutagenesis and the discriminatory phenotypic characteristic of this strain is its temperature sensitivity [8]. The MS1 vaccine strain has been commercially released recently, it was obtained by spontaneous mutation during *in vitro* passages and it does not require NAD for its growth [9]. The re-isolation of the vaccine strains and the examination of the phenotypic characteristics are time-consuming [17]. Genotyping based on the *vlhA* gene is used worldwide for the molecular differentiation of *M*. *synoviae* strains, but this method was proved to be unreliable in the discrimination of the MS-H vaccine strain [11,18–19]. High resolution melt-curve analysis based assay was developed to identify specific SNPs in the *obg* gene in order to distinguish the ts^+^ MS-H vaccine strain from its ts^-^ re-isolates and field strains [11]. In our previous study, the MS-H1 and MS-H2 MAMAs were designed to differentiate the SNPs in the *obg* gene to enable rapid, simple and cost efficient detection of the MS-H vaccine strain [12]. The purpose of the current study was to develop a DIVA-test for the recently released MS1 vaccine strain, which can be applied simultaneously with the assays used for the differentiation of the MS-H vaccine strain. Thus melt- and agarose-MAMAs were designed to identify the SNP at the nt11 in the HIT family protein coding gene with the same thermocycling profile as the MS-H1 and MS-H2 assays. The developed assay provides a reliable tool in routine diagnostics for the differentiation of the MS1 strain, as it is a sensitive and specific test and it can be performed directly on the clinical samples and in laboratories even with basic real-time PCR or conventional PCR equipment. The MS1 and MS-H vaccine strain specific MAMAs could efficiently support control programs against *M*. *synoviae* infections in one rapid, convenient and cost efficient step.
